# Parallel and private generalized suffix tree construction and query on genomic data

**DOI:** 10.1186/s12863-022-01053-x

**Published:** 2022-06-17

**Authors:** Md Momin Al Aziz, Parimala Thulasiraman, Noman Mohammed

**Affiliations:** Department of Computer Science, University of Manitoba, 66 Chancellor Drive, Winnipeg, Manitoba, R3T2N2 Canada

**Keywords:** Privacy-preserving Queries on Genomic Data, Outsourcing Genomic Data on Cloud, Parallel Construction of Generalized Suffix Tree, Reverse Merkle Tree

## Abstract

**Background:**

Several technological advancements and digitization of healthcare data have provided the scientific community with a large quantity of genomic data. Such datasets facilitated a deeper understanding of several diseases and our health in general. Strikingly, these genome datasets require a large storage volume and present technical challenges in retrieving meaningful information. Furthermore, the privacy aspects of genomic data limit access and often hinder timely scientific discovery.

**Methods:**

In this paper, we utilize the Generalized Suffix Tree (GST); their construction and applications have been fairly studied in related areas. The main contribution of this article is the proposal of a privacy-preserving string query execution framework using GSTs and an additional tree-based hashing mechanism. Initially, we start by introducing an efficient GST construction in parallel that is scalable for a large genomic dataset. The secure indexing scheme allows the genomic data in a GST to be outsourced to an untrusted cloud server under encryption. Additionally, the proposed methods can perform several string search operations (i.e., exact, set-maximal matches) securely and efficiently using the outlined framework.

**Results:**

The experimental results on different datasets and parameters in a real cloud environment exhibit the scalability of these methods as they also outperform the state-of-the-art method based on Burrows-Wheeler Transformation (BWT). The proposed method only takes around 36.7s to execute a set-maximal match whereas the BWT-based method takes around 160.85s, providing a 4× speedup.

**Supplementary Information:**

The online version contains supplementary material available at (10.1186/s12863-022-01053-x).

## Introduction

In today’s healthcare system, human genomics plays a vital role in understanding different diseases and contributes to several domains of our healthcare system. Over the years, genomic data have given us new areas of research such as genomic or personalized medicine and genetic engineering. Therefore, with the recent technological advancements, we can store millions of genomes from thousands of participants alongside their medical records. Today, medical professionals from different geo-location can utilize these massive interconnected datasets to study disease-phenotype associations or susceptibility to certain diseases [1].

Furthermore, due to the reducing cost of genome sequencing, the recruitment for corresponding research or studies is getting popular [2]. There are several consumer products that appeared over the past year such as Ancestry.com, 23AndMe.com. Nevertheless, these real-world applications share one major computation on human genome data which is *String Search* [3]. Informally, string search in this context denotes the locations and often the presence of a query genome, representing similarity in terms of our genomic markup. Therefore, a high degree of similarity in genomic data can indicate the likelihood of similar physical traits or ancestry.

On the other hand, due to the unique nature of human genomes, privacy aspects of this sensitive data is surfacing over the last decade [4]. Therefore, the current privacy regulations do not allow genomic datasets to be publicly available without any formal application and require due diligence from the researchers [5]. This can attribute a delay to the scientific discoveries depending on sensitive genomic data and the participants’ medical records [3].

Therefore, employing privacy-preserving techniques while performing sensitive queries on a genomic dataset is an important research area. This field has attracted the cryptographic community in general where several theoretically proven private frameworks are being investigated [3, 6]. Specifically, the massive scale of genomic data and computational complexity of the queries have made this area challenging where we would protect the privacy of the participants while providing a timely response from the privacy-preserving computations.

In this paper, we target suffix trees, specifically Generalized Suffix Tree (GST) which can be employed to perform several search operations on genomic data [7]. Firstly, we construct GST in parallel (published in the conference version [8], which is later extended with privacy-preserving string query techniques using GST indexing. It is important to note that building a suffix tree efficiently and in parallel is a well-studied area and not our primary contribution. Instead, we target GSTs which can represent a genomic dataset containing multiple participants [9] where we employed distributed and shared memory architectures for parallel construction. Distributed architecture considers multiple machines with completely detached memory systems, connected with a network. Our mechanism utilizes the global memory in this case harnessing the parallel power of the several cores available.

Primarily, we propose privacy-preserving methods to perform arbitrary string queries on the genomic dataset. The proposed method relies on a hash-based scheme combined with cryptographic primitives. With two different privacy-preserving schemes, we demonstrate that the proposed methods provide a realistic execution time for a large genomic dataset. The contributions of this paper are:

- The novelty of this work lies in the proposed private query execution technique that incorporates a hashing mechanism (Reverse Merkle Hash) over a tree structure that additionally serves as a secure index allowing several string search operations. We further extend this method’s security with Garbled Circuit [10] where the researcher’s inputs are deemed private as well.
- Initially, we propose a GST construction mechanism using different memory models using parallel computations.
- Efficiency of the GST index along with the privacy-preserving queries are tested with multiple string searches. Specially, we analyze speedups altering the number of processors, input dataset size, memory components and different indexing.
- As reported in our earlier version [8], experimental results show that the proposed parallel construction can achieve ∼4.7× speedup in comparison to the sequential algorithm for a dataset with 1000 sequences and each sequence with 1000 nucleotides (with 16 processors).
- Our privacy-preserving query mechanism also demonstrates promising results as it only takes around 36.7 seconds to execute a set-maximal match in the aforementioned dataset. Additionally, we compared with a private Burrows-Wheeler Transform method [11] which takes around 160.85 seconds giving us a 4× speedup. Our secure query method is also faster than Sotiraki et al.’s [12] which needed 60 seconds under the same setting.

The paper is organized as follows. Methodology section describes the proposed methods for parallel GST construction and privacy-preserving queries. Experimental results are shown and discussed in Experimental results and analysis section as potential limitations and future works are added as well. The related works and background techniques are described in the Supplementary Materials. Finally, Conclusion section presents the conclusion of the paper. It is noteworthy that the parallel GST construction is available in the conference version [8] which is summarized in Methodology and Experimental results and analysis sections as well.

## Methodology

As we fist build the **GST in parallel** prior to the **private execution** of different queries, the proposed methods are divided into two major components. Nevertheless, the architecture of the problem and proposed method are summarized below. Notably, the parallel GST construction is also available in our conference version [8]:

### Problem architecture

**Fig. 1:**
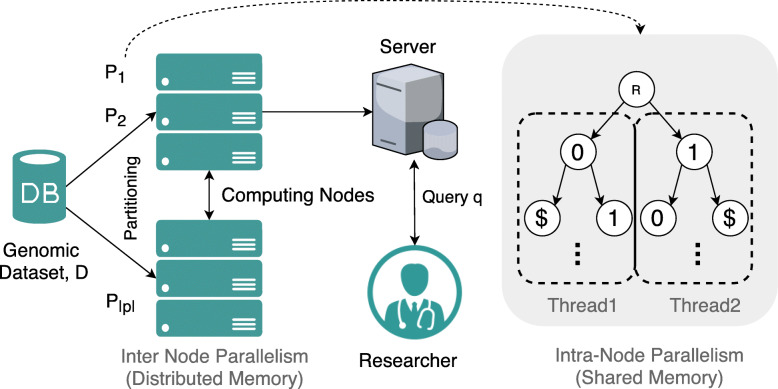
Computational framework of the proposed method where the data owner holds the genomic dataset and constructs the GST in parallel on a private computing cluster (one-time preprocessing). The GST is then outsourced securely to the Cloud Server (CS) where the query *q* from researcher is executed in a privacy-preserving manner

The architecture consists of three entities: a) Data Owner, b) Cloud Server and c) Researchers as outlined in Fig. 1. Here, data owner collects the genomic dataset *D*_*n*×*m*_ where string queries *q* are executed by any researcher. The queries are handled by an intermediary cloud server as the data owner generates a Generalized Suffix Tree (GST) and stores it privately on the cloud. The background on GSTs are available on the supplementary material. We assume that the researchers have limited computational power since they are interested in a small segment of the dataset $\mathcal {D}$. Also, researcher have no interaction with the data owner as all query operations are handled by the cloud server. In summary, the proposed method presented in this article has two steps: a) constructing the GST in parallel, and b) executing *q* with a privacy guarantee over the data.

#### Parallel GST construction [8]

Parallel GST construction will first evenly partition the genomic data into different computing nodes. Here, we employ two memory environments— a) distributed, and b) shared. Distributed memory setting has the machines interconnected via a network where they contain *mutli-core* processors and fixed-size memory (RAM). The multiples cores in these processors also have the physical memory namely shared memory.

We propose the memory distribution to address the large memory requirement while constructing the trees. For example, *n* sequences with *m* genomes may take at least *nm* memory resulting in any real-world genomic dataset overfitting the memory. Therefore, this issue gave the motivation to build GST for a targeted genomic dataset in a *distributed memory setting* [8].

#### Private storage and queries

After constructing the GST in parallel in a private cluster, the resulting GST is stored in a offshore semi-trusted cloud system. The utility of a commercial cloud service is motivated by its low cost and higher storage requirement from GSTs built on genomic data. Furthermore, cloud ser- vice provides a scalable and cost-effective alternative to the procurement and management of required infrastructure costs, which will primarily handle queries on genomic data. As shown in Fig. 1, the researchers only interact with the cloud server, which contains the parallel constructed GST.

However, using a third-party vendor for storing and computing sensitive data is often not permissible as there have been reports of privacy attacks and several data leaks [3]. Therefore, we intend to store the genomic data on these cloud servers with some privacy guarantee and execute corresponding string queries alongside. Specifically, our privacy-preserving mechanisms will conceal the data from the cloud server; in case of a data breach, the outsourced genomic data cannot be traced back to the original participants. Further details on the threat model are available in Privacy model.

#### String Queries *q*

We considered different string queries to test the privacy-preserving methods proposed based on GSTs and other cryptographic scheme (check supplementary materials). The four queries discussed here are incrementally challenging while the inputs to these queries will be the same $\mathcal {D}$. Since we are considering a dataset of size *n*×*m* haplotypes, $\mathcal {D}$ will have {*s*_1_,…*s*_*n*_} records where *s*_*i*_∈[0,1]^*m*^. The query needs to be less than the number of genomes (1≤|*q*|≤*m*).

##### **Definition 1**

**(Exact Match-EM)** For any arbitrary query *q* and genomic dataset $\mathcal {D}$, exact match will only return the record *x*_*i*_ such that *q*[0,*m*]=*x*_*i*_[0,*m*] where *m* is the number of nucleotides available on each genomic sequence in $\mathcal {D}$.

##### **Example 1**

A haplotype dataset, $\mathcal {D}$ is presented in Table 1 of size *n*×*m*, where *n*=5 and *m*=6. For a query, *q*={1,0,0,0,1,0}, exact queries according to the aforementioned Definition 1 will perfectly match the first row *x*_*i*_; hence the output set for this input *q* will be the first sequence in *X*.

**Table 1:** Sample haplotype data representation where *s*_*i*_∈{0,1}^*m*^ are the different positions on the same sequence

#	*SNP*_1_	*SNP*_2_	*SNP*_3_	*SNP*_4_	*SNP*_5_	*SNP*_6_
1	1	0	0	0	1	0
2	1	1	1	0	1	0
3	1	1	0	0	0	1
4	0	1	0	1	1	0
5	0	1	0	1	0	1

##### **Definition 2**

**(Exact Substring Match-ESM)** Exact substring match should return the records *x*_*i*_ such that *q*[0,|*q*|−1]=*x*_*i*_[*j*_1_,*j*_2_], where *q*[0,|*q*|−1] represents the query and *x*_*i*_[*j*_1_,*j*_2_] is a substring of the record *x*_*i*_ given *j*_2_≥*j*_1_ and *j*_2_−*j*_1_=|*q*|−1.

##### **Example 2**

For an exact substring match query, we need a query sequence, where |*q*|<*m*. For *q*={1,1,1}, the output of the query (according to Definition 2) should contain the second row as the query sequence, *q* is present in the dataset, $\mathcal {D}$ as a substring.

##### **Definition 3**

**(Set Maximal Match-SMM)** Set maximal match, for the same inputs will return the records *x*_*i*_, which have the following conditions:

1. there exists some *j*_2_>*j*_1_ such that *q*[*j*_1_,*j*_2_]=*x*_*i*_[*j*_1_,*j*_2_];
2. *q*[*j*_1_−1,*j*_2_]≠*x*_*i*_[*j*_1_−1,*j*_2_] and *q*[*j*_1_,*j*_2_+1]≠*x*_*i*_[*j*_1_,*j*_2_+1], and
3. for all *i*^′^≠*i* and *i*^′^∈*n*, if there exist $j_{2}^{\prime } > j_{1}^{\prime }$$q[j_{1}^{\prime },j_{2}^{\prime }]=x_{i}[j_{1}^{\prime },j_{2}^{\prime }]$ then it must be $j_{2}^{\prime } -j_{1}' < j_{2} - j_{1}$.

##### **Example 3**

A set maximal match can return multiple records that partially matches the query. For *q*={1,1,0,1}, it will return the records {2,3,4,5} from $\mathcal {D}$ as outputs since they have 1101,110,101,101 substrings, respectively.

##### **Definition 4**

**(Threshold Set Maximal Match-TSMM)** For predefined threshold *t*, TSMM will report all records following the constraints from SMM (Definition 3) and *j*_2_−*j*_1_≥*t*.

##### **Example 4**

Inheriting from Definition 4, we have an additional parameter, threshold, *t* which determines the number of mismatches allowed in the output sequences. For a query *q*={1,0,1,1} and threshold *t*≥3, the output will be {2,4,5} since the second and fourth record have 101 starting from positions 3 and 2, respectively and the fourth sequence completely matches the query from position 2.

### Parallel GST construction [8]

In this section, we summarize the proposed techniques to construct the GST in parallel from our earlier work [8]. These approaches fundamentally differ in partitioning and agglomeration according to the PCAM (Partitioning, Communication, Agglomeration and Mapping) model [13]:

#### Data partitioning scheme [8]

The memory location and the number of distributed computing nodes allowed us to employ two data partitioning scheme: Horizontal and/or Vertical [8]: **Horizontal** partitioning makes a different group of sequences according to the computational nodes or processors available. Each node will receive one group and perform the GST construction in parallel. For example, for *n*=1000 and *p*=4, the data is split into 4 groups, each with |*n*_*i*_|=25 sequences. Each processor node *p*_*i*_ will build GST individually on |*n*_*i*_| sequences of *m* length. This process is done in parallel and does not require any communication. In supplementary materials, we discuss an example of our horizontal partition scheme for two nodes (*n*=*p*=2).

**Vertical** partitioning scheme cuts the data along the genomes or columns and follows a similar mechanism mentioned above. However, splitting across the columns presents an additional complexity upon merging which is discussed in Distributed memory model [8].

**Bi-directional** scheme performs data partitioning along the rows and columns, combining earlier approaches. It is noteworthy that this partition scheme only works with four or more processors or *p*≥4. For example, with *n*=100,*m*=100 and *p*=4, each processor will get a *n*_*i*_×*m*_*i*_=50×50 records for their computations.

#### Distributed memory model [8]

**Fig. 2:**
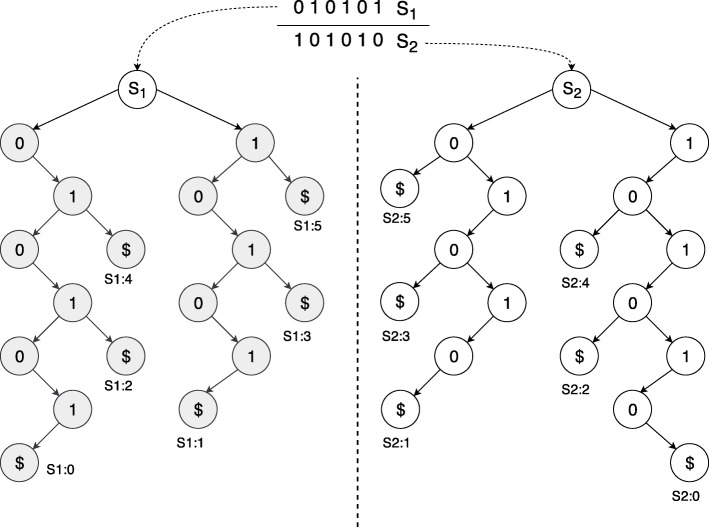
Uncompressed Suffix Tree (Trie) construction

The interconnected nodes receive the partitioned genomic data and start building their individual GSTs in parallel. For example, *p*_0_,*p*_1_…,*p*_|*p*|_ nodes will create *GST*_0_,…,*GST*_|*p*|_ suffix trees in parallel. It is noteworthy that, the underlying algorithm for constructing the GSTs is linear employing Ukkonen’s algorithm [14], regardless of the partitioning mechanism. Once the build GST phase is completed, these nodes start the network activity by sharing their GSTs for the merge operation:

Figure 2 shows a GST construction for horizontally partitioned data. Here, two different suffix trees are presented on the left side, nodes coloured in grey and white. The merged version of these trees is on the right. It is important to note that, the merge operation should not create any duplication of nodes at any particular level. However, for the other partitioning schemes (vertical and bi-directional), we will need to perform an extra step where the datasets are divided against the column (*m*_*i*_<*m*).

**Fig. 3:**
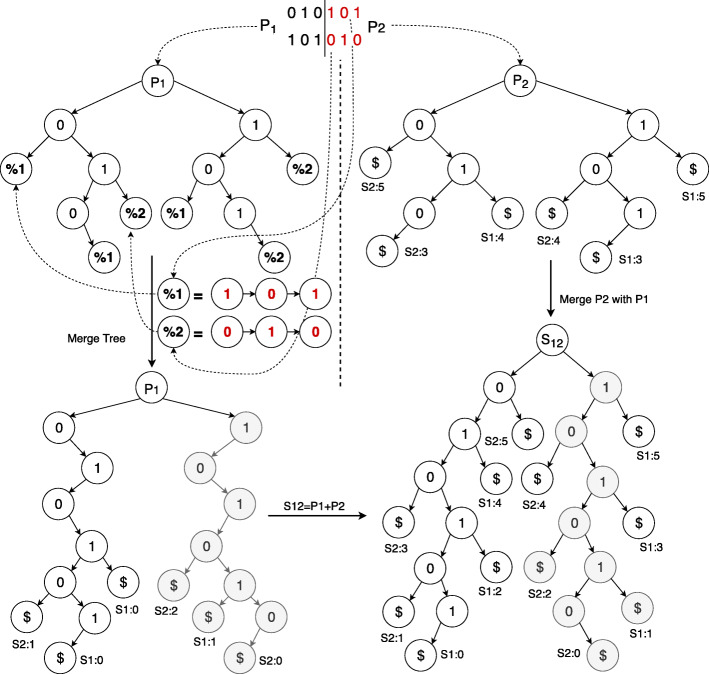
Vertical partitioning with path graphs (*%*1,*%*2) merging [8]

Figure 3 shows this step where GSTs are constructed for S1,S2 ={010101,101010} with a setting of *n*=2,*p*=2,*m*=6. Here, the first node *p*1 takes {010,101} as input whereas *p*2 operates on {101,010}. Here, the GST from *p*1 does not have the full sequence and needs to account for the tail-end suffixes that are generated over at *p*2. Therefore, we added different end characters to *p*1’s suffix trees, representing the future addition.

Based on this end character, a merge operation happens for all cases where sequences were partitioned across the columns, without the last genomes *m*_*i*_<*m*. However, suffix trees from the tail-end sequences (*m*_*i*_=*m*) can be described as linear or *Path Graphs*. For example, in Fig. 3, 101 and 010 are represented as *%*1,*%*2 where both are linear nodes or path graphs. We add these *%*1,*%*2 path graphs to the suffix trees on *m*_*i*_<*m* without any duplication. Finally, the trees on *p*1 and *p*2 are merged according to the previous method, following the horizontal partitioning scheme.

#### Shared memory model [8]

In summary, the distributed memory model had multiple instances with completely different memory environments where the GSTs were constructed. Now, these instances also have multiple CPU cores accessing a global memory (RAM). In this shared memory model, we utilize these in processor cores and perform a parallel merge operation.

Our genome data consist of a fixed alphabet set considering the nucleotides available (A, T, G, C). We use this property here proposing an *Intra*-node parallel operation using the shared memories among the cores. Here, the number of children is always fixed due to the fixed alphabet size, we propagate the operations into multiple cores. For example, one core only handles the suffixes with 0 at the beginning (or root) whereas another one takes only the 1 branch. Figure 2 depicts this operation where *p*1 and *p*2 constructs individual GSTs from {01,0101,010101} and {1,101,10101}. Then, the output GSTs are merged, avoiding duplicates and added to the final GST’s root. Notably, due to the limited main memory in this shared environment, we cannot process arbitrary large datasets only using this method.

#### Merging GSTs [8]

Since GSTs are constructed in multiple processors and memory environments, we need to merge them for the final GST representing the whole genome dataset. Here, the merge operation takes multiple GST as input and produces a single tree without any duplicate node on a single level (Definition 5). Formally, for *p* processors, we need to merge |*p*| GSTs to create the final *GST*; *GST*=*GST*_0_+…+*GST*_|*p*|_. We use the technique discussed in Shared memory model [8] treating the 0 and 1 children of the root into separate cores. Notably, the branches from 0 or 1 child of root do not have any common edges between them. Therefore, we can perform merges in parallel availing the intra-node parallelism.

##### **Definition 5**

(Merge GSTs) Given two suffix trees *T*_1_ and *T*_2_ from two sequences *S*1 and *S*2 with *m* length, the leaf nodes of the merged tree *T*_12_ will contain all possible suffixes of *S*1:*i* and *S*2:*ii*∈[1,*m*].

**Fig. 4:**
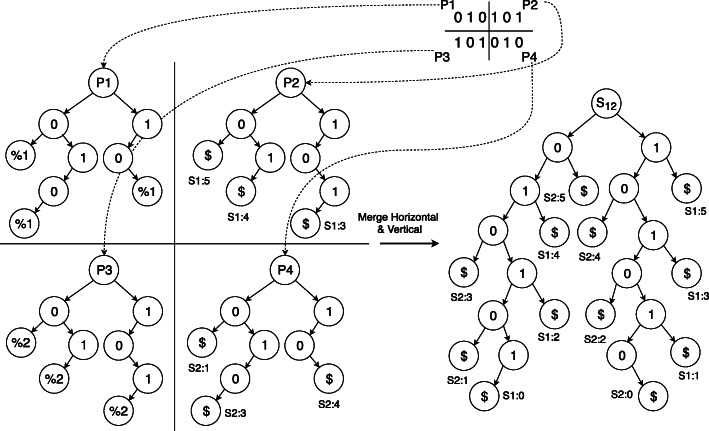
Bi-Directional partitioning scheme where data is separated into both rows and columns and merged using the shared memory model [8]

An example of the merge operation is shown in Fig. 4 depicting a bi-directional partition and merging afterwards. Notably, merging any branch to another is a sequential operation. Here, different threads cannot operate simultaneously for the integrity of the tree or avoid race conditions. Nevertheless, the intra-node parallelism can be extended according to the number of cores available. For example, rather than only considering 0 and 1 branches, it can take 11,10,01,00 branches.

#### Communication and mapping [8]

In our proposed mechanism, the computing nodes get a continuous segment of genomic data on which they construct their GSTs. The final GST in any node is saved in a file system which is later communicated through the network with the other participating nodes. We chose the merge operation to occur between the closest nodes or with the least latency present. As an example, for Fig. 4*p*3*p*4 will share their GSTs with *p*1,*p*2, respectively. Both *p*1,*p*2 will perform the merge operation in parallel while the GSTs were received as files. Here, the primary reason behind using files or external memories is solely for the memory requirements from large genomic datasets which can create a memory overflow for a single node.

### Privacy preserving query execution

In this section, we discuss the mechanisms that allow privacy preserving queries on suffix trees.

#### Merkle tree

Merkle tree is a hash-based data structure which is often used as a data compression technique [15]. Here, the data are represented as leaf nodes of a binary tree and they are hashed together in a bottom-up fashion. The individual node values are determined from its children as they are concatenated and hashed with any cryptographic hash function (i.e., MD5, SHA-2 [16] etc.). For example, the parent *A* of leaf nodes with value 0 and 1 will denote *A*=*h*(*h*(0) || *h*(1)) where *h* is a hash function with fixed output size *k* as *h*:{0,1}^∗^→{0,1}^*k*^. Similarly, if its sibling is denoted by *B*, then their parent will have *C*=*h*(*h*(*A*) || *h*(*B*)) where || represents concatenation.

#### Reverse Merkle tree (RMT)

**Fig. 5:**
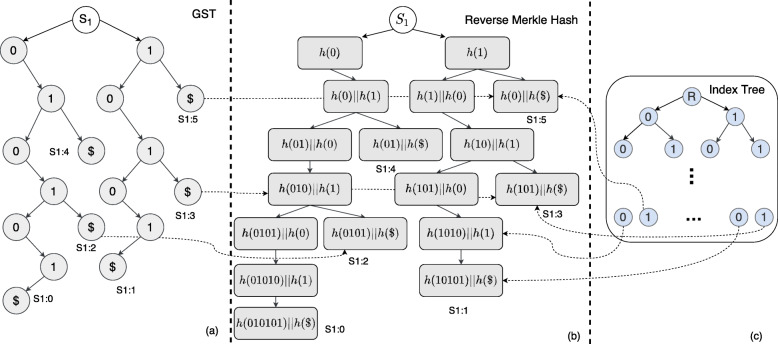
Reverse Merkle Hash for Suffix Tree on *S*1=010101 where we hash the value of each node in a top-down fashion

In this work, we utilize a reverse of the Merkle Tree hash where the data is hashed in a top-down fashion. For example, a child node will have the hash value *A*=*h*(*P* || *h*(0)) where 0 and *P* is the hash value of the node and its parent, respectively. The sibling will have *B*=*h*(*P* || *h*(1)), analogously as shown in Fig. 5b. We initialize the root’s hash value with a random value namely SALT for additional security which is mentioned in Privacy preserving query execution.

Here, as the GST is constructed in parallel, we hash the content of the individual nodes alongside the SNP values. The hash values are passed down to the children nodes and added with their hashed SNP value. In Fig. 5, we show the example of a reverse hash tree for the sequence *S*1=010101. Here, in each node, we take the hash of the parent node and add it to the hash of that node’s value. Notably, in Fig. 5, we write *h*(*AB*) to replace *h*(*h*(*A*) || *h*(*B*)) in short. The leaf nodes will also have the position of the suffix appended together with the nucleotide value (represented as $ in Fig. 5b.

The rational behind using the reverse Merkle tree is to represent the suffixes using the hash values for faster matching. Here, the hash values on the leaf nodes represent the corresponding suffixes of that edge in the GST. For example, the longest path in Fig. 5 will represent *S*1:0 and contains the hash for suffix 010101. We also keep the position of the suffix alongside the hash values. These leaf hash values are kept separately for incoming queries which accelerate the search process as we describe it in Privacy preserving query execution.

##### **Definition 6**

(Reverse Merkle Tree) For a sequence *S*=*s*_1_*s*_2_…*s*_*m*_ and a deterministic hash function *h*:{0,1}^∗^→{0,1}^*k*^, the Reverse Merkle Tree (RMT) will produce a hash output *h*(*S*)=*h*(…(*h*(*h*(*s*_1_) || *h*(*s*_2_)) || …)).

##### **Example 5**

For a sequence *S*=0110, RMT will initially produce the hash *h*(*s*_1_) where *s*_1_=0. It will proceed to the next character *s*_2_=1 and concatenate both the hash outputs. However, *h*(*s*_1_) || (*s*_2_) doubles the size of the fixed bit hash output which is then hashed again to make it of the same size. *h*(*h*(*s*_1_) || (*s*_2_)) is then concatenated with *h*(*s*_3_) as RMT represents the final output *h*(*h*(*h*(*h*(0) || *h*(1)) || *h*(1)) || *h*(0)).

#### Cryptographic hash function

The cryptographic function employed to hash the values in each node is quite important. As there are multiple hash functions available (i.e., MD5, SHA-1 [16], etc.), they ultimately serve a similar purpose. These functions provide a deterministic, one-way method to retrieve a fixed bit size representation of the data. Therefore, it can also be considered as a compression technique that offers a fixed size for arbitrary large genomic sequences or suffixes.

We utilized MD5 as an *example* function in our implementations as it was executed on every node as described in Reverse Merkle tree (RMT). Here, it is important to consider the size of the hashed values as MD5 provides a fixed 128-bits output. Using another hash function with better collision avoidance or more security (i.e., SHA-1) may result in longer (256 bits) hash values, which will increase the execution time linearly in order of the bit size. Nevertheless, MD5 is given as an example that can be replaced with any cryptographic hash function.

#### Suffix tree storage

One of the major limitations of Suffix Trees is the number of nodes and the storage they require for longer input sequences. In the worst case, a sequence of length *m* will have *m*+1 unique suffixes. The number of suffixes also increases along with the values of sequence and genomes within (*n*,*m*). For example, *m* bi-allelic SNPs from one sequence can create 2^*m*+1^−1 nodes on the suffix tree.

The content of these nodes is hashed according to the aforementioned Reverse Merkle Tree method. Due to the size of the resulting tree and its dependency on the size of the sequence, we utilize file-based storage, in place of the main memory. Here, all operations on the suffix tree, construction and queries are run on physical files, which are later outsourced to the external semi-trusted computational environment. We next discuss the privacy model and the privacy-preserving outsourcing mechanism.

#### Privacy model

The primary goal of the proposed method is to ensure the privacy of the data (located on the GST) in an untrusted cloud environment. Therefore, we expect the cloud to learn nothing about the genomic sequences beyond the results or patterns that are revealed from the traversal. Note that the proposed method do not guarantee the privacy derived from the query results as it might be possible for the researchers to infer private information of an individual using the query results. The proposed secure techniques do not defend the genomic data against such privacy attacks, where researchers may act maliciously. Nevertheless, we discuss some preventive measures using differential privacy in Discussion.

The privacy assumption for the cloud service provider (CS) is different as we adopt the semi-honest adversary model [17]. We assume that CS will follow the implicit protocols but may attempt to retrieve additional information about the data from the underlying computations (i.e., logs). This is a common security definition, and realistic in a commercial cloud setting since any cloud service providers comply with the user agreement and cannot use/publish the stored data without lawful intervention. Furthermore, in case of a data breach on the server, our proposed mechanism should protect the privacy of the underlying genomic data. In addition, the system has the following properties: a) CS does not collude with any third party or researchers to learn further information, b) in case of an unwanted data breach on CS, the stored GST (or genomic data) does not reveal the original genomic sequences, and c) Researchers are assumed honest as they do not collude with other parties to breach the data.

Formally, let researcher and cloud server be *P*_1_ and *P*_2_, respectively. *P*_2_ stores a private database $\mathcal {D}'$ as *P*_1_ wants to execute a string function $f(q, \mathcal {D}')$ based on a query string *q*. For example, this function can be any string query defined in Definitions 1, 2, 3pdefsmm and 4. The privacy goal of the targeted method will be to execute $f(q, \mathcal {D}')$ in a way that *P*_1_ and *P*_2_, both are unaware of each other’s input, but only knows the output of *f*. We assume that *P*_2_ is semi-honest as it does not deviate from the protocol. Furthermore, no polynomially bounded adversary can infer the sensitive genomic data from outsourced $\mathcal {D}'$ if it gets compromised.

#### Privacy-Preserving outsourcing

As the GST is constructed in parallel in a private cluster, the resulting suffix tree is stored (or outsourced) in a commercial cloud server (CS). The researchers will present their queries to this CS, and CS will search on the GST for the corresponding queries. For example, if we consider the four queries from String Queries *q*, each will warrant a different number of searches throughout the outsourced GST.

Since we intend to ensure the privacy of the genomic data in an untrusted environment, we remove the plaintext nucleotide values from the GST replacing them with their Reverse Merkle hash value according to Definition 6. For example, GST in Fig. 5a will be hashed in a top-down fashion where the leaf nodes will contain the sequence number and corresponding suffix position.


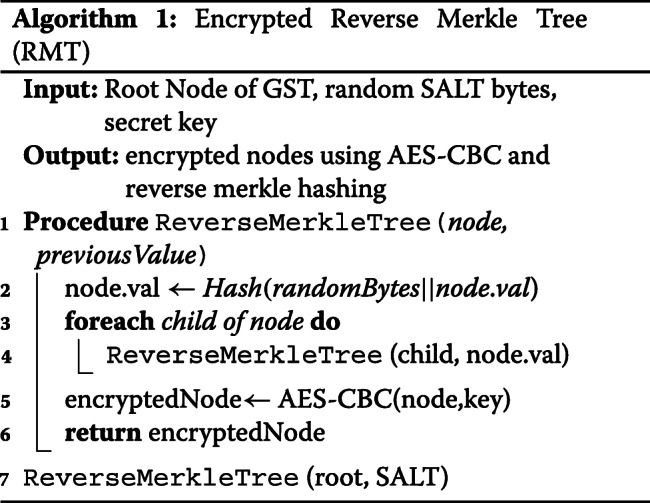


Since a genomic dataset will only have limited input characters (A, T, G, C), hashing them individually will always produce the same output. As a result, CS (or any third party) can infer the hashed genomic sequences. Therefore, to protect the privacy of the data, we utilize two methods: a) A random byte array is added to the root of the GST, kept hidden from the CS, and b) the final hash values are encrypted with Advanced Encryption Standard (AES) in the block cipher mode (AES-CBC) prior to their storage.

As the one-way public hash function reveals the genomic sequence due to its limited alphabet size, we need to randomize the hash values so that no adversary can infer additional information. Such inference is avoided with a standard random byte array, namely SALT. Here, the root of the GST (Fig. 5a) contains a SALT byte array which is never revealed to CS. As this SALT array of the root node is appended to its children nodes, it will cascadingly alter all the hash values downstream making them appear random.

For example, while generating Fig. 5b from a, the left and right child of root *S*1 will contain the value *h*(*SALT* || *h*(0)) and *h*(*SALT* || *h*(1)), respectively. For simplicity, the random SALT byte can be assumed to be of the same length as of the hash function output, *k* (128 random bits for MD5). Since CS does not know these random *k* bits, it will need to brute force through the 2^*k*^ possible values which is exponential in nature. Since the hashing is also done repeatedly, it can prove to be challenging to infer meaningful information from the RMT hash tree for an arbitrarily long genomic dataset. Notably, the SALT bytes are shared with the researcher as it is required to construct the queries as well.

**Fig. 6:**
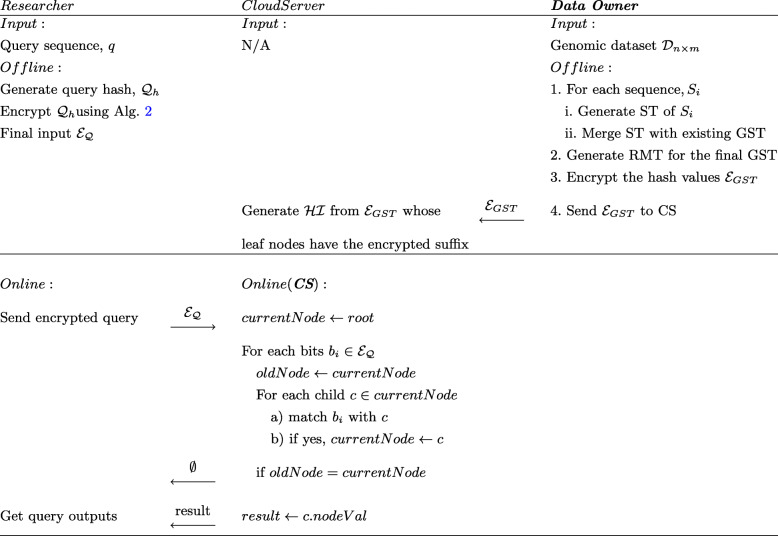
The search protocol of our proposed solution for Exact Match (Definition 1). Data owners are offline after sharing the encrypted GST to CS as the researchers and CS only need to be online for search operation. The encrypted query $\mathcal {E}_{\mathcal {Q}}$ are send to CS and matched against the $\mathcal {HI}$ for the final result

To further improve the security, these individual hash values are also encrypted with AES-CBC with 128 bit keys. This AES mode requires an random Initialization Vector (IV) which is also shared with the researcher but kept hidden from CS. This encryption provides an additional layer of security in an unlikely event if CS gets compromised. The encrypted hash values will be randomized and should prevent further data leakage. The procedure to get the Encrypted Reverse Merkle tree is described in Algorithm 1. In summary, the output from data owner to CS will be the encrypted GST, $\mathcal {E}_{GST}$ where every node value is encrypted. We demonstrated the process in Fig. 6.

Therefore, according to our privacy model in Privacy model, the RMT containing the encrypted hash values of the original dataset is safe to transfer over to a semi-honest party [17]. As we also assume the CS to be honest-but-curious [17], it will follow the assigned protocols and will not attempt any brute force attacks on the hashed values. However, under any data breach, the proposed encrypted tree will suffer the same limitations of symmetric encryption. Notably, some of them can be avoided by using asymmetric encryption or separate secret keys for different heights or depth of the GST which will strengthen the security; we discuss this in Discussion.

It is important to note that the size of the suffix tree is an important factor to consider when deciding on the underlying cryptosystem. We picked the symmetric encryption scheme, AES partially due to this reason as it will not increase the size of the hash output. For example, the output from MD5 for every suffix tree node will be 128 bits. These 128 bits are later encrypted with AES-CBC which represents the final content stored on the suffix tree nodes. Here, the encrypted hash values do not increase the size of the content.


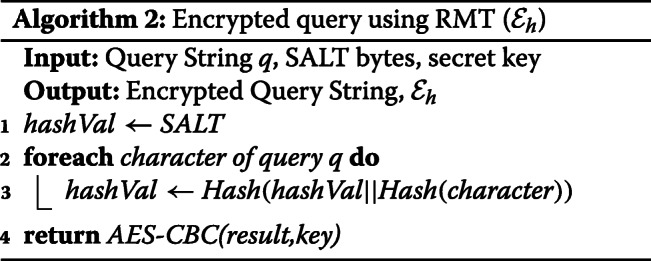


#### Privacy-Preserving query execution

The four queries mentioned in String Queries *q* will be executed over the AES-CBC encrypted RMT hash values as outlined in Reverse Merkle tree (RMT). These hash values compress the nucleotides available on each edge to a fixed number of bits (size of the hash) and offer an advantage when searching over the whole GST.

##### Hash Index ($\mathcal {HI}$):

Prior to the query, CS creates another intermediary index on the encrypted hash values from $\mathcal {E}_{GST}$. Since our hash function will always provide a fixed sized output (in bits) for each node, a binary tree can effectively speed up the search which is constructed over the symmetrically encrypted bits of $\mathcal {E}_{GST}$. For example, MD5 will always output the same 128-bis for the same SALT and series of nucleotides using RMT. Encrypting these fixed size values with AES-CBC with the same key will produce ciphertexts which can later be utilized for searching as the researchers will come up with the same ciphertexts for any matching query.

The output from the AES-CBC bits are kept in a binary tree having a fixed depth of 128 (from root to leaf) as we use 128 bit encryption. Here, the leaf nodes will point towards the hash value or the nodes appearing on the RMT. We name this B-tree as $\mathcal {HI}$ as it replaces an exhaustive search operation on GST (outlined in Fig. 6). Notably, we added the positions of the suffixes from GST into the $\mathcal {HI}$ using the end character $ symbol which was appended to the genomic sequences. This positional value (i.e., $0 representing *S* : 0) contained the starting index of the suffix which was necessary for all queries with a targeted position.

We can demonstrate the efficacy of $\mathcal {HI}$ for the Exact Match (EM) query as defined in Definition 1. Here, the researcher initiates the query as s/he can have one or multiple genomic sequences to search for in the dataset $\mathcal {D}$. The researcher constructs the hash representation of the query using the secret key and random byte array (SALT) that was employed to make the GST stored in the CS. For example, if the query is 010101, then the query hash will be: $\mathcal {Q}_{h} =h(\ldots h(h(SALT\, \vert \vert \,h(0))\, \vert \vert \,h(1))\ldots)$. Later, it will be encrypted with the key $\mathcal {E}_{\mathcal {Q}}= \mathcal {E}(\mathcal {Q}_{h},key,IV)$ and sent to CS for matching. The procedure to retrieve $\mathcal {E}_{\mathcal {Q}}$ is briefed in Algorithm 2.

CS will search for this $\mathcal {E}_{\mathcal {Q}}$ in the fixed size ($\mathcal {HI}$) first. If the hash exists on the B-tree, CS returns the leaf node that $\mathcal {HI}$ is referencing. Here, only the leaf nodes of $\mathcal {HI}$ keep a reference of the Reverse Merkle Tree nodes which is sent as the final result to the researcher (in case of a match). For a mismatch, we will not have a node on $\mathcal {HI}$ for the query hash, resultingly, do not need to check GST anymore.

###### **Lemma 1**

For a hash function with fixed output size *k*, Exact Match (Definition 1) will require a worst-case runtime of $\mathcal {O}(\log k)$ for any arbitrary query.

In Lemma 1 we consider the output size of the hash function for simplicity as AES will produce the same number of bits as inputs. Nevertheless, we can extend the method for EM (same runtime as Lemma 1) to execute the rest of the queries. For example, a substring Match can be an extension of EM where we will consider a query length, |*q*| smaller than the sequence length (<*m*) and only allowing exact matches of |*q*| length. This is also possible employing $\mathcal {HI}$ which represents the strings residing in a GST.

Similarly, for the Set Maximal Matching (SMM-Definition 3), the researcher and CS perform an iterative protocol. The researcher initially searches for the whole query following the same protocol from Fig. 6 on the $\mathcal {HI}$ leading to the GST residing in CS with the specific position. For a mismatch, it reduces the query length by one and iterates the search until there is a match. The worst-case running time for such operation will be in order of $\mathcal {O}(\vert q\vert \log k)$. PVSMM (Definition 4) is an extension of the same protocol where we have a threshold constraint which further reduces the computations to $\mathcal {O}(t\log k)$ given *t*>|*q*|.

#### Hiding query access pattern

Garbled Circuit (GC) allows our two-party, CS and the researcher to execute a secure protocol between themselves which ensures their input privacy guarantee. In our proposed method, the encrypted $\mathcal {HI}$ does the major search operation as it is outsourced on CS. However, the input from the researcher is not hidden from CS. Such access pattern for an arbitrary query might reveal additional information which we avoid using this method.

In this GC protocol, the hash values are represented as binary strings and matched against the $\mathcal {HI}$ using the oblivious transfer technique. The researcher produces the reverse Merkle hash value $\mathcal {Q}_{H}$ according to algorithm 2. Here, the query sequence will be a Path as each node will have only one child. Each query node will denote the corresponding nucleotide in the specific position. For example, the root will have the first nucleotide (lowest position) as its child and the leaf node will be the last position of the sequence.

We perform the reverse Merkle hashing on such a Path graph where the leaf node will represent the final query hash value. The root of this path will contain the same secret SALT used to construct the reverse Merkle tree. The resulting fixed-length hash value is then matched with the binary tree, $\mathcal {HI}$ on the CS through a GC protocol. Notably, the size of the query hash and the height of $\mathcal {HI}$ are the same as we use the same hash function.

Here, CS and the researcher goes to a fixed round interaction where each bit from the query hash serves an input from the researcher while CS puts the bit value from $\mathcal {HI}$. For example, in the first round, the researcher puts the first bit of the query hash in the GC protocol. CS randomly picks a child from the root of the $\mathcal {HI}$ (0 or 1) and sets it as its input for GC. The circuit utilized here is an XNOR operation between these two input bits which results in 1 only if the input bits are the same. Importantly, the input bits are not revealed to any party while the output is only available for CS. If their input bits matches then CS proceeds with the currently selected node’s children or take its sibling. There are only 2 nodes at each level as $\mathcal {HI}$ is a B-Tree.

CS then sends a request to match the next bit and both parties repeat the procedure until a mismatch. Here, a mismatch of the bits denotes that the CS does not have such hash value on the GST, hence the query sequence is not available in the dataset. In this case, CS returns that there are not sequence on dataset $\mathcal {D}$ matching the query. For a match on the leaf node, CS returns the encrypted suffix sequence that are referenced at the $\mathcal {HI}$ leaf nodes. Since the four queries can essentially reduce to matching the hash values on the query path and $\mathcal {HI}$ (Lemma 1), we do not discuss the corresponding GC protocols for each query in detail. Shortly, SMM or TSMM will require the researcher to remove the nucleotides from his/her query and iterate the GC protocol for each query edits.

In our proposed method, the researcher and CS can guess each other’s inputs as it is a XNOR circuit on the hash outputs. However, the input query sequence and the data on GST is kept hidden from both parties in case of a mismatch. We argue that under the targeted privacy model (Privacy model), the resulting sequences and the query can be public to both parties. Nevertheless, we also considered a non-iterative mechanism to perform the search which operated on the full binary tree of $\mathcal {HI}$ (input from CS). Here, the complete encrypted query hash $\mathcal {E}_{\mathcal {Q}}$ from the researcher is an input on the GC while CS inputs $\mathcal {HI}$. This method matches each hash bits obliviously and only outputs the matching suffix; avoiding the disclosure from the in-between matches. However, it incurred a longer execution time which is further discussed in our limitations in Discussion.

## Experimental results and analysis

Before discussing the findings, we will describe the implementation and underlying dataset details:

### Datasets and evaluation

Since the proposed method is scaled and evaluated against different parameters, we use randomly distributed synthetic datasets. We generate different datasets with {*n*,*m*}∈{200,300,…,1000} and name them such as *D*_*n*×*m*_. We agree that genomic data of *n*,*m* in millions will portray the true benefit of our proposed parallel constructions, but due to our computational restrictions, we limited our experimental settings [18]. However, we argue that larger datasets will denote the same trend in terms of execution time as we increase the parallel computational power. Our implementations are publicly available in [19].

### Suffix tree construction speedup [8]

We measure the suffix tree construction speedup according to the dataset size (*n*,*m*), number of processors *p* and different memory models—distributed, shared and hybrid. The distributed or shared model does not employ intra-node or inter-node parallel operations whereas the hybrid method utilizes both. It is important to note that the results for the parallel GST construction is also available in [8] which we summarize here.

Tables 2 and 3 contains the execution time for all three partitioning scheme—horizontal, vertical and bi-directional. We also report the results from the three memory architecture varying the number of processors *p*={2,4,8,16}. Notably, the sequential or serial execution is denoted by the *p*=1 case which is a plain Ukkonen’s algorithm [14].

**Table 2:** Execution time (in minutes) for Horizontal and Vertical partition scheme with processors *p*={1,2,…,16}

Data	Serial	Distributed				Shared				Hybrid			
	1	2	4	8	16	2	4	8	16	2	4	8	16
Horizontal Partitioning													
200	0.08	0.23	0.09	0.09	0.10	0.14	0.05	0.04	0.03	0.14	0.07	0.05	0.05
300	0.27	1.04	0.23	0.2	0.23	0.38	0.15	0.11	0.08	0.37	0.16	0.12	0.12
400	0.59	2.03	0.55	0.38	0.38	1.18	0.35	0.21	0.2	1.12	0.31	0.23	0.25
500	1.53	3.14	1.32	1.06	1.01	2.27	0.57	0.36	0.28	2.09	0.52	0.38	0.41
1000	14.55	16.23	8.34	6.31	6.09	17.38	5.56	3.27	2.28	17.14	4.18	3.12	3.08
Vertical Partitioning													
200	0.08	0.19	0.08	0.05	0.03	0.16	0.07	0.04	0.02	0.14	0.05	0.03	0.02
300	0.27	0.56	0.28	0.17	0.09	0.48	0.22	0.16	0.08	0.39	0.13	0.10	0.06
400	0.59	1.41	1.05	0.36	0.16	1.44	1.01	0.34	0.19	1.21	0.32	0.21	0.13
500	1.53	3.07	1.49	1.08	0.37	3.18	1.49	1.08	0.36	2.35	0.58	0.40	0.24
1000	14.55	25.24	12.25	9.06	5.20	22.56	13.11	7.2	4.37	18.22	6.31	4.49	3.10

**Table 3:** GST construction time (in seconds) using bi-directional partition scheme with processors *p*={1,4,8,16} [8]

Data	Serial	Distributed			Shared			Hybrid			
	1	4	8	16	4	8	16	4	8	16	32
200	4.8	94.2	90	87	43.8	42.6	38.4	70.8	73.2	75.6	1.51
300	16.2	121.8	107.4	106.2	72	48.6	43.2	88.8	75	75.6	1.37
400	35.4	168.6	148.2	124.8	102.6	54	57.6	114	87	96	1.36
500	91.8	231.6	151.8	154.8	145.2	76.2	62.4	146.4	103.8	105	1.36
1000	873	1135.2	428.4	291.6	856.8	202.2	154.8	635.4	312	214.2	1.36

Table 2 shows that GST building time for smaller datasets are almost the same for all memory models and experimental settings. However, the execution time difference starts to be clearer as we increased the dataset size (*n*,*m*). For example, *D*_200×200_ needed 0.08 mins on the serial execution (*p*=1) whereas *D*_1000×1000_ required 14.55. The distributed model needed 6.09 minutes showing the added network complexity and operations required by inter-node communication. The hybrid memory model performed better taking only 3.08 mins with 16 processors.

Interestingly, for these smaller datasets, the shared memory model outperforms the other memory settings. Unlike the distributed model, the shared architecture requires no network connectivity as it splits the work into different cores. It required the lowest time in all settings, around 2.28 minutes with 16 processors. However, it did successfully process larger dataset, greater than 1000 nucleotides and genomes. Since the memory and processors in a shared model is fixed which can be extended in distributed setting by adding new machines, larger datasets will require the later approach.

In Table 2, we show the results from vertical partitioning, having an extra step of path graph addition. This addition is not present on the horizontal partitioning. This additional step increased the execution time, taking 25.24 minutes to process *D*_1000×1000_, compared to 16.23 mins with the horizontal partitioning. The bi-directional partitioning results are shown in Table 3. Compared to the prior two data partitioning schemes, the tree build cost is reduced here as there are smaller sub-trees to join in this case. For example, with four processors (*p*=4) and *n*=*m*=1000, each processor will get an input of 25×25 genome dataset, leading to four subsets of 100×25 and 25×100 partitions for vertical and horizontal schemes, respectively.

In Table 4, we report the execution time for individual operations: tree building, path graph addition and tree merge. Here we report the maximum time for each operation from each run since they were run in parallel. It is noteworthy that these operations are the building blocks for the execution time posted in Tables 2 and 3. Table 5 summarizes the speedup results for dataset *D*_1000×1000_. Speedup is defined as *T*_*par*_/*T*_*seq*_ where the shared model performed better than the distributed one. However, the distributed model’s results are comparable for *p*>2 settings. Notably, the shared architecture could not process the dataset, *D*_10,000×10,000_ due to memory constraints and excluded from the results. This limitation was not preset for both distributed and hybrid setting as they were able to construct the GST.

**Table 4:** Maximum Execution time (in seconds) of Tree Building (TB), Add Path (AP) and Tree Merge (TM) for dataset, *D*_1000_

*p*	Horizontal			Vertical			Bi-directional		
	TB	AP	TM	TB	AP	TM	TB	AP	TM
4	113.35	-	70.02	292.97	2.7	66.8	4.01	0.37	3.85
8	47.38	-	85.4	138.87	2.9	61.1	0.62	0.16	1.8
16	15.6	-	98	64.4	3.2	57.6	0.12	0.07	1.2

**Table 5:** Results on the speedup for dataset, *D*_1000×1000_ for all memory models and partitioning schemes with processors *p*={2,4,8,16}

Method	Distributed			Shared			Hybrid		
	4	8	16	4	8	16	4	8	16
Horizontal	1.19	1.61	2.80	1.11	2.02	3.33	2.31	3.24	4.69
Vertical	1.74	2.31	2.39	2.62	4.45	6.38	3.48	4.66	4.72
Bi-directional	0.77	2.04	2.99	1.02	4.32	5.64	1.37	2.80	4.08

One of the limitations of the proposed framework is the size of the resulting suffix tree. Since the node contents are hashed and encrypted, it also increases the memory requirements as we utilized file-based memory to handle queries. For example, for a dataset of {500×500,1000×1000,2000×2000,5000×5000} takes around {109,433,1754,11356} megabytes of storage space. Notably, this storage accounts for the hashed tree and the AES-CBC encrypted node values on the Merkle tree. Furthermore, we opted to experiment with a relational database (MySQL) to save the encrypted tree, which is detailed in our code repository [19].

### Query execution

#### Experimental setup

In this section, we analyze and discuss the execution time of the four queries in private and non-private settings as defined in String Queries *q*. We utilized an Amazon EC2 cloud server (specification g4dn.xlarge) in Oregon, US as the cloud server and the researcher was located in Winnipeg, Canada. The average network latency between the CS and the researcher was around 49ms. The key components of the result analysis are as following:

1. Execution time for all queries with worst-case inputs,
2. Effect of dataset size and query length
3. The impact of GST and $\mathcal {HI}$, and
4. The runtime comparison between hashing and GC

We targeted the worst-case input queries as it will highlight the maximum execution time for each type of query. For example, for exact matches (EM), we randomly picked a query sequence from the dataset. As any mismatch on the $\mathcal {HI}$ will forcefully reduce the computations, we chose to pick available sequences for Query 1. For SMM and TSMM Queries (3 and 4), we preferred a random query sequence which was not present in the dataset. As for a mismatch, SMM (and TSMM) will redo the search altering the query sequence. This will show the maximum execution time required. Alternatively, if we picked a sequence from the dataset (similar to EM), it was not necessary to traverse the $\mathcal {HI}$ and it will output the same execution time as EM.

Therefore, our targeted four queries can essentially be reduced to EM. For example, we do not discuss the exact *substring* matches in this section as it took the same time as the EM. We also limit the execution time for two datasets *D*_1000_ and *D*_500_ as the data dimension will increase the size of the GST. Therefore, we examine the scalability issues with different query lengths |*q*|∈{300,400,500} and (*n*,*m*)∈{(1000,1000),(500,500)}.

#### Execution Time for GST (w/o privacy)

Initially, we analyze the execution time of the targeted queries on plaintexts without any privacy guarantee in Table 6. Here, we only execute the queries on the generalized suffix tree (GST) as they are outsourced on CS and simulate the researcher on the same server to avoid the random network latency. The execution time from the Table 6 clearly shows that longer query sequences (i.e., |*q*|=500) require more time than smaller queries. As we are searching on the suffix tree, our GST indexing presents a runtime linear in the query length of |*q*|. Notably, GST allowed us to remove the runtime dependency with the number of sequences or nucleotide (*n* or *m*) which is often higher for genomic datasets.

**Table 6:** Exact Matching, SMM and TSMM (Query 1, 3 and 4) using GST considering different datasets and query lengths (time in milliseconds)

Query Length |*q*|	*D*_1000_			*D*_500_		
	EM	SMM	TSMM	EM	SMM	TSMM
300	0.5	140	94	0.3	80	70
400	0.5	140	150	0.4	130	120
500	0.6	210	220	0.5	190	180
1000	1.1	680	720	-	-	-

One interesting observation here is the scalability property of GST on different sized datasets. As we considered two different datasets *D*_1000_ and *D*_500_ with *n*,*m*={1000,500}, it seems that the runtime does not increase significantly. Ideally, traversing the GSTs from *D*_1000_ or *D*_500_ for a query should not be different but the increased number of nodes on memory adversely affects the query execution.

#### Execution time for $\mathcal {HI}$ (with privacy)

Since the query length |*q*| can also be arbitrarily large, we reduce its impact on execution time by employing $\mathcal {HI}$. This index $\mathcal {HI}$, built on the GST allows us only to search up to the hash output length |*H*| rather than |*q*|. We see its effect in Table 7, as for different |*q*|’s, the execution time for EM did not increase which was the opposite for plaintext GST as shown in Table 6.

**Table 7:** Secure Exact Matching (EM), SMM and TSMM (Query 1, 3 and 4) using $\mathcal {HI}$ considering different datasets and query lengths (time in milliseconds). QP, GC, |*q*| denotes query processing time, Garbled Circuit, and Query Length respectively

|*q*|		Reverse Merkle Hash with $\mathcal {HI}$						GC		Shimizu et al. [11]	
		*D*_1000_			*D*_500_			*D*_1000_	*D*_500_	*D*_1000_	*D*_500_
	QP	EM	SMM	PVSMM	EM	SMM	PVSMM	EM	EM	SMM	SMM
300	0.79	41.4	11599	2862	37.1	11100	2761	63246	63583	50358	43163
400	0.84	43.9	15337	3901	36.9	15385	3760	63194	62639	64867	55609
500	0.9	42.7	18563	4836	37.2	18477	4875	63439	62048	70754	67965
1000	1.58	45.2	36761	9368	-	-	-	63391	-	160854	

Since we considered the worst-case inputs (non-matching query sequences) for SMM and PVMM, both types of queries required more matching requests on the cloud server. These iterative query executions increased the runtime incrementally. The effect of the dataset size is also analogous with our earlier argument as the time vary slightly for different sized datasets. We do not show the results for SMM over the garbled circuit as they required over an hour each on the worst-case inputs.

We also benchmark with recent work from Shimizu et al. [11] which utilized positional Burrows-Wheeler Transformation with Oblivious Transfer (OT-secure protocol) for input privacy. From the results in Table 6, it is evident that our Merkle hash along with $\mathcal {HI}$ provides a 4× speedup compared to the earlier work as it takes 160.85 seconds to execute a set maximal match on *D*_1000_ (our method required 36.76s). However, since this benchmarking method only used OT rather than more expensive GC operations, it was faster than the GC protocol. The implementations from Sotiraki et al. [12] was not available publicly which is why we could not add it to our benchmarking results.

## Discussion

In this section, we discuss some of the core results, limitations and some potential future works as well:

**Parallel construction of GST:**

GST provides an efficient index to the genomic data which can be used in many string-based search queries, fundamental to the utility of genomic data. However, the best sequential algorithm is linear to the sequence length which can prove to be significant for a large dataset with longer genomic sequences *n*,*m*. Therefore, constructing such an expensive tree-based data structure is handled by the proposed parallel mechanism, which is required to be executed only once while pre-processing any dataset.

**Storage complexity of GST:**

On contrary, we use a file-based GST for two fundamental reasons: a) higher storage requirement for the suffix tree, and b) fixed main memory in comparison to persistent disk memory. This also warrants the usability of cloud servers, which offer less-expensive storage solutions. Here, GST warrants an expensive storage cost as the number of suffixes increases linearly in order of the length of the sequence (*m*). For example, a genomic sequence of length *m* has *m*+1 suffixes which increases for increasing values of *n*,*m*. Also, for *m* genomes (bi-allelic SNPs), in the worst case, it can create 2^*m*+1^−1 nodes on the suffix tree. Resultingly, we incorporate another fixed-size index $\mathcal {HI}$ on GST, which acts as the principal component while searching and can fit into the main memory.

**Privacy guarantee from encrypted hash value:**

The privacy of the data relies on the symmetric AES cryptosystem along with the random SALT bytes kept on the root node of Reverse Merkle Hashing. We did not use any asymmetric public-key encryption scheme due to the resulting ciphertext size expansion. Nevertheless, the recently improved homomorphic encryption schemes might be beneficial in this task and provide additional security guarantee [20, 21] which is an interesting future work.

**Privacy Guarantee from GC:**

In our proposed method (Hiding query access pattern), GC plays its part in matching the bit values of the query hash and node values on $\mathcal {HI}$. Here, the researcher and CS are unaware of each party’s inputs unless there is a match. However, it still reveals the encrypted query for a query sequence that exists on $\mathcal {HI}$. This could be avoided with a more rigorous GC protocol where the whole query $\mathcal {E}_{\mathcal {Q}}$ and $\mathcal {HI}$ will be taken as inputs. However, searching the whole query obliviously were not computationally feasible and we did not report it here. This process can be efficient with leveled execution of the searching on $\mathcal {HI}$ which can be investigated in the future.

**Output privacy:**

To protect the genomic data against any malicious researchers, we can perturb the outputs from CS with some privacy guarantee. One method to attain output privacy is by adding noise to the query results, and these techniques have been studied in the literature such as anonymization [22], differential privacy [3]. However, we did not opt for these strategies as they will thwart the exact results from any query and validity is quintessential in any scientific research. The realistic model in genomic research also assumes the researchers to be honest as they adhere and understands the privacy requirements of genomic data.

## Conclusion

Executing string queries on genomic data is not a new research area; however, a privacy-preserving approach for string queries has received little attention in the literature. The primary contribution of this paper is a hash-based mechanism to outsource and execute privacy-preserving queries on genomic data. Due to the expensive construction operation, a parallel generalized suffix tree building is proposed that utilizes both distributed and shared processing capabilities and external memory. The proposed parallel constructions and privacy-preserving query techniques can also be generalized for other data structures (e.g., prefix trees [23], PBWT [11]) and thus can be useful for different genomic data computations. We also analyzed the performance using different datasets and sample string queries. Experimental results show that the proposed methods are more efficient than the state-of-the-art techniques for string query execution.

## Supplementary Information

**Additional file 1** Supplementary Materials

## Data Availability

Please check [19] for the open-source implementation and the required data (https://github.com/mominbuet/ParallelGST).

## References

[1] Cerami E, Gao J, Dogrusoz U, Gross BE, Sumer SO, Aksoy BA, Jacobsen A, Byrne CJ, Heuer ML, Larsson E (2012). The cBio cancer genomics portal: an open platform for exploring multidimensional cancer genomics data. AACR.

[2] Schwarze K, Buchanan J, Taylor JC, Wordsworth S (2018). Are whole-exome and whole-genome sequencing approaches cost-effective? a systematic review of the literature. Genet Med.

[3] Aziz MMA, Sadat MN, Alhadidi D, Wang S, Jiang X, Brown CL, Mohammed N. Privacy-preserving techniques of genomic data—a survey. Brief Bioinforma. 2017. 10.1093/bib/bbx139 PMC6585383 29121240

[4] Bonomi L, Huang Y, Ohno-Machado L (2020). Privacy challenges and research opportunities for genomic data sharing. Nat Genet.

[5] Naveed M, Ayday E, Clayton EW, Fellay J, Gunter CA, Hubaux J-P, Malin BA, Wang X (2015). Privacy in the genomic era. ACM Comput Surv (CSUR).

[6] Akgün M, Bayrak AO, Ozer B, Sağıroğlu MŞ (2015). Privacy preserving processing of genomic data: A survey. J Biomed Inf.

[7] Mahdi MSR, Al Aziz MM, Mohammed N, Jiang X (2021). Privacy-preserving string search on encrypted genomic data using a generalized suffix tree. Inform Med Unlocked.

[8] Aziz MMA, Thulasiraman P, Mohammed N, Martín-Vide C, Vega-Rodríguez MA, Wheeler T (2020). Parallel generalized suffix tree construction for genomic data. Algorithms for Computational Biology.

[9] Farach M, Ferragina P, Muthukrishnan S (1998). Overcoming the memory bottleneck in suffix tree construction. Proceedings of the 39th Annual Symposium on Foundations of Computer Science.

[10] Yao AC-C (1982). Protocols for secure computations. FOCS, vol 82.

[11] Shimizu K, Nuida K, Rätsch G (2016). Efficient privacy-preserving string search and an application in genomics. Bioinformatics.

[12] Sotiraki K, Ghosh E, Chen H (2020). Privately computing set-maximal matches in genomic data. BMC Med Genomics.

[13] Foster I (1995). Designing and Building Parallel Programs: Concepts and Tools for Parallel Software Engineering.

[14] Ukkonen E (1995). Online construction of suffixtrees. Algorithmica.

[15] Merkle RC. Method of providing digital signatures. Google Patents. 1982. US Patent 4,309,569.

[16] Gupta P, Kumar S (2014). A comparative analysis of sha and md5 algorithm. Architecture.

[17] Lindell Y, Pinkas B (2009). A proof of security of yao‘s protocol for two-party computation. J Cryptol.

[18] Computing Resources. www.cs.umanitoba.ca/computing. Accessed 4 Dec 2019.

[19] Aziz MMA. Implementation for Parallel Private GST. https://github.com/mominbuet/ParallelGST. Accessed 25 Mar 2022.

[20] Gentry C, et al.Fully homomorphic encryption using ideal lattices. In: Stoc, vol 9: 2009. p. 169–78.

[21] Morshed T, Alhadidi D, Mohammed N. Parallel linear regression on encrypted data. In: 2018 16th Annual Conference on Privacy, Security and Trust (PST). IEEE: 2018. p. 1–5.

[22] Wang S, Mohammed N, Chen R (2014). Differentially private genome data dissemination through top-down specialization. BMC Med Inf Dec Making.

[23] Chen L, Aziz MM, Mohammed N, Jiang X (2018). Secure large-scale genome data storage and query. Comp Methods Prog Biomed.

