# Supplementary material for: Parallel and private generalized suffix tree construction and query on genomic data
Source: BMC Genom Data. 2022 Jun 17;23:45. doi: 10.1186/s12863-022-01053-x (PMC9206251; doi:10.1186/s12863-022-01053-x)
Supplement: Supplementary file 1 — Additional file 1 Supplementary Materials [file 12863_2022_1053_MOESM1_ESM.pdf]

Supplementary Document: Parallel and Private Generalized Suffix Tree Construction and Query on Genomic Data

Md Momin Al Aziz, Parimala Thulasiraman, and Noman Mohammed

In this Supplementary Material, we discuss the related works in the area and the preliminaries for the interested readers. The data and implementations are available in github [1]. Please check for more details.

I. Related Works

Since we target two different research area concerning genomic data, we discuss the related works separately below:

A. Privacy-preserving String Search

There are different types of string search functions targeted on genomic data using different cryptographic protocols. Table I summarizes some of the the existing approaches for privacy-preserving searches where we show the high-level difference among them.

Apart from these cryptographic computation approaches, there has been several works on publishing genomic data with theoretical privacy guarantee. Chen *et al.* [2] proposed a differentially private [3] mechanism to publish n-grams with variable-lengths. These n-grams can be used for count queries or mining string patterns. However, due to the Laplace noise utilized, the outputs will have inaccuracies which we do not have in this work.

Secure exact string search has been a popular since the realization of the privacy aspects of human genomic data. In 2003, Atallah *et al.* [4] proposed a dynamic programming algorithm to compare two sequences using homomorphic encryption. Other contemporary approaches utilized finite automata with Recursive Oblivious Transfer (ROT) to solve the DNA searching problem [5]. Troncoso *et al.* [6] proposed a similar approach for approximate string matching requiring (amortized) linear time. Sudo *et al.* [7] presented a unique algorithm employing secure wavelet matrix and additive homomorphic encryption to search for substrings in logarithmic time. Using Garbled Circuits (GC) and tree-based index, Mahdi *et al.* [8], [9] proposed count queries on genomic data which relied on exact string matching. In this work, we avoid the expensive GC protocols with Reverse Merkle Hashing.

Secure exact substring search targeting a specific position is a related but a different problem where the SNP position plays an important part. In this paper, we proposed a secure model to find a match between query and dataset sequences, where a starting position can be specified. In 2016, Ishimaki *et al.* [10] addressed this problem with a look-up table constructed from positional Burrows-Wheeler transform (PBWT) and utilized Fully Homomorphic Encryption (computation under encryption).

MMA. Aziz, P. Thulasiraman and N. Mohammed are with the Department of Computer Science, University of Manitoba, Winnipeg, Canada. E-mail: [azizmma,,noman]@cs.umanitoba.ca

TABLE I: Related works in different privacy-preserving genomic string search

Work	Exact	Substring	Set-Maximal	Query Pos
Atallah *et al.* [4]	✓	✓	✗	✗
Sudo *et al.* [7]	✓	✓	✗	✗
Ishimaki *et al.* [10]	✓	✓	✗	✓
Shimizu *et al.* [11]	✗	✗	✓	✓
Sotiraki *et al.* [15]	✗	✗	✓	✓
Our Work	✓	✓	✓	✓

Set-maximal match is another important problem that is employed in genealogy queries popular in ancestry searching. Shimizu *et al.* [11] introduced a secure variable length prefix or suffix matching on SNP genomic sequences. The authors utilized Positional Burrows Wheeler Transform (PBWT) as proposed by Durbin *et al.* [12] for preprocessing the raw genomic data (alternatively, we employed the GSTs). To ensure the privacy, we utilized the reverse Merkle hash with random SALT, whereas the authors used the Recursive Oblivious Transfer (ROT) with additive homomorphic encryption. Yamada *et al.* [13] utilized a fully (and somewhat) homomorphic encryption schemes to formulate a string search framework. However, the query lengths were only selected from $[5, 25]$ taking a maximum of 80 seconds. In comparison, our method only takes around 40 seconds for a query size of 1000.

In iDASH 2019 competition [14], there was a separate track for secure set-maximal matching due to its popularity on ancestry applications. The winning solution was proposed by Sotiraki *et al.* [15] using Goldreich-Micali-Wigderson (GMW) protocol [16]. Here, the problem required two parties (data owner and researcher) and needed a private evaluation of XOR, AND and NOT operation between them. In comparison, in this paper, we propose a secure mechanism to outsource genomic data to public cloud and execute similar queries. Our secure query method is also faster as it takes around 12 seconds for a query size 300 and database size of 1000×1000 where Sotiraki *et al.* takes around 60 seconds.

B. Parallel GST Construction

Suffix tree construction is a fairly mature and well-studied problem as there have been multiple works which are shown

TABLE II: Design-level comparison of previous and our method in parallel GST construction

Work	Parallelism model		Disk-based	GST
	Distributed	Shared		
TDD [19]	✗	✗	✓	✗
TRELLIS [20]	✗	✗	✓	✗
Wavefront [21]	✓	✗	✓	✗
ER_A [22]	✓	✗	✓	✗
PCF [23]	✓	✗	✗	✗
DGST [24]	✓	✗	✓	✓
Our Work	✓	✓	✓	✓

in Table II. Since Generalized Suffix Tree of a large genomic dataset does not fit a sizeable memory, there have been attempts to construct the tree in a file system [17]. These disk-based suffix trees usually store the individual subtrees on file similar to our approach [18]. For example, Tian *et al.* [19] showed a different suffix tree merging method *ST-Merge* using the Top-Down Disk (TDD) Algorithm.

Wavefront [21] and its successor ER_A (Elastic Range) [22] both targeted disk-based and parallel approach to construct suffix trees. However, these works only considered a suffix tree and distributed memory model, whereas, in this work, we propose a hybrid method and GST. Comin and Farreras [23] proposed Parallel Continuous Flow (PCF) which efficiently distributes the lexical sorting process into multiple processors. Analogous to this work, Shun and Belloch [25] also proposed a parallel construction scheme utilizing *cilk* (shared memory) in 2014. Flick and Alura [26] proposed another distributed in-memory suffix tree construction solving the All Nearest Smaller Values (ANSV) problem. However, both works targeted suffix trees whereas GST can contain a large number of sequences which is more complicated and at the same time more useful.

There have been some Hadoop MapReduce based solutions to create suffix trees [27]. Li *et al.* [28] proposed a spark implementation of ER_A to construct GST where it was parallel to the number of sequences. In a recent work in 2019, DGST [24] offered a 3× speed up with such data-parallel platform which is better than the state-of-the-art method ER_A [22]. Nevertheless, it did not employ the shared or hybrid model as we performed better with 4× speedup. Notably, Mišić *et al.* report speedup up to 6× utilizing parallelism from Graphics Processing Units (GPUs) [29]. However, we do not use any specialized hardware and could not benchmark as their implementations are unavailable.

In this paper, we target an out-of-core GST construction using distributed processors along with their multiple cores in parallel. We utilize a simple data partitioning scheme to demonstrate that our methods are comparable to these proposed methods in terms of speed-ups. Nevertheless, this parallel construction is not the primary contribution as it served as a gateway for the privacy-preserving queries on genomic data.

II. Preliminaries

In this section we discuss the necessary background for the paper including the underlying data, problem definitions and briefly overview the generalized suffix trees.

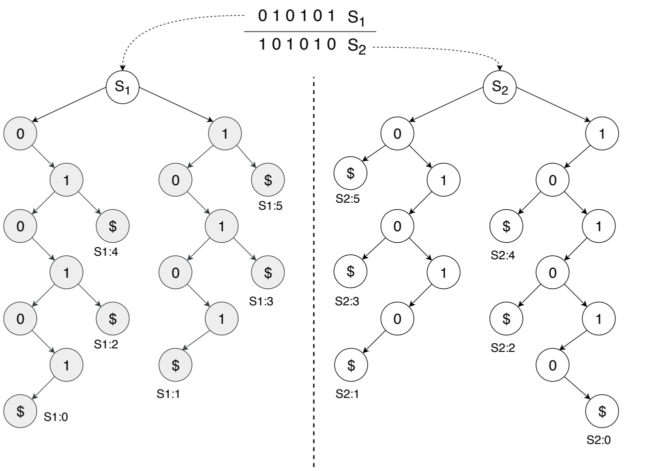

Fig. 1: Uncompressed Suffix Tree (Trie) construction

A. Haplotype Data

Our genomic data inside the chromosomes consist of four nucleotide values represented by A, T, G and C. However, each location of individual chromosome mostly have two possible values which are named as alleles and these locations are named bi-allelic. These bi-allelic genome data can be represented as a binary sequence containing 0's and 1's where 0 and 1 denotes the reference and observed allele, respectively. Multi-allelic such as tri-allelic sites are rare (around 2% in human genome [30]) as it can have three different alleles present at a single location.

Therefore, we consider the bi-allelic genomic data which is also called haplotype data, where each allele (or position) on the chromosome is inherited from a single parent. In other words, in one specific location, we can only perceive two variations for such a dataset; therefore, we utilize a binary representation. However, our proposed method is not limited to such binary representation and generalizable over any dataset with a fixed character domain.

Formally, a S be a haplotype sequence with m alleles such as $S = s_1 s_2 \dots s_m$ over a fixed size alphabet $\sum \in \{0, 1\}$. A substring of S is another string $S : i^j = s_i s_{i+1} \dots s_j$ where $1 \leq i \leq j \leq m$. A suffix is a specialized substring where $S : i = s_i s_{i+1} \dots s_m$ with length $m - i$.

B. Generalized Suffix Tree

Suffix Trie and Tree:: Trie (from re**trie**val) is a data structure where each data point is placed in the vertex of a tree. Here, the edges represent the relation of one data to the other. In our problem scenario, each nucleotide of a sequence can be seen as the other data points or vertices of a Trie. We can differentiate two notable categories of Trie in literature: a) Prefix or b) Suffix. Specifically, we are interested in suffix tries in this paper, though the proposed method is generalizable towards prefix trie/tree construction as well.

Definition 1. (Trie [31]) a trie is a rooted tree where each node represents a symbol from the alphabet $\sum$ (except root) and no two sibling (children of the same node) share a common symbol.

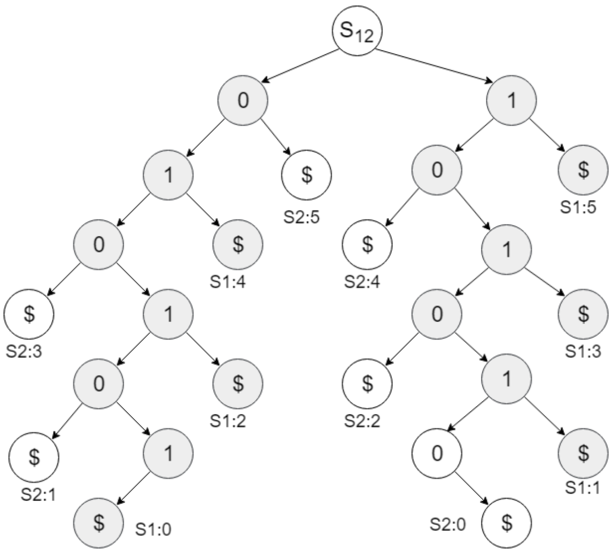

Fig. 2: GST from Figure 1 where gray and white vertices are from `S1` and `S2`, respectively

Similarly, the Suffix Tries from sequence S is a rooted tree which contains all possible suffixes of S at its leaf nodes. Notably, the memory requirements of the such suffix trie is significant as it needs $\mathcal{O}(m^2)$ for m characters. However, Suffix Trees are a compressed version of their Trie counterpart as it reduces the quadratic size to a linear one. For example, if a single vertex has only one child on a suffix trie, they are joined and denoted as a single vertex on the Suffix Tree. In Figure 1, we show two suffix tries from sequences, `S1`$=010101$ and `S2`$=101010$ respectively. For `S1`, we consider all possible suffixes such as $S1 : i = [1, 01, 101, 01010, 10101, 010101]$ st $i \in \{1, |S1|\}$and construct the suffix tree.

Definition 2. (Suffix Tree [32]) A Suffix Tree for an m character string S is a Trie (definition 1) consisting of exactly m leaf nodes. Each node (except root and leaves) have at least two children where the edges represent a non-empty substring of S and no two sibling can have the same starting character. Finally, the m strings obtained from m leaf nodes should represent every suffix of $S : i$ where $i = 1, 2 \ldots, m$.

The suffix tree will also represent the end of the sequence with a special end character (`$`). For example, the suffix 01 (left Figure 1-`S1`) has an end character with label `S1:4` which denotes the sequence number and the start position of the suffix. Here, a node is labeled as `Sx:y` *s.t.* $x \in \{1, n\}$ and $y \in \{0, m-1\}$ for n sequences of m length.

Generalized Suffix Tree (GST): Generalized Suffix Tree is a collection of suffix trees (following definition 2) constructed for multiple sequences. Here, we merge two suffix trees `S1` and `S2` from Figure 1 and construct `S12` in Figure 2. Fundamentally, there are no difference in constructing GST as we need to build individual suffix tree per sequence and merge them afterwards. Thus, the runtime for one GST construction depends on these suffix tree construction and size. For example, the traditional Ukkonen algorithm to build the suffix tree has a linear runtime $O(m)$ for m length sequences [33]. Therefore, to build the GST for n sequences with m characters requires time $\mathcal{O}(nm)$.

C. Garbled Circuit

A Garbled Circuit (GC) is a constant round protocol that allows any function to be securely computed between multiple parties. Defined in 1982 [34], it can best be explained by 'The Millionaire Problem', where two people (preferably millionaires) want to determine who is richer without revealing their exact wealth to any party. They can initiate a secure protocol (GC) between them, and the resulting boolean value will denote which party's value (or wealth) is greater.

GC is a cryptographic protocol, where one party (generator) generates the garbled circuit for a particular computation and holds secret mappings before the inputs and garbled values. The other party (evaluator) evaluates this circuit using the cryptographic keys. Here, the execution time of this protocol is proportional to the complexity of the underlying computations or, specifically, the depth of the circuit.

References

[1] Md Momin Al Aziz. Implementation for Parallel Private GST. https://github.com/mominbuet/ParallelGST. Accessed 2021-09-08.

[2] Rui Chen, Gergely Acs, and Claude Castelluccia. Differentially private sequential data publication via variable-length n-grams. In *Proceedings of the 2012 ACM conference on Computer and communications security*, pages 638–649, 2012.

[3] Cynthia Dwork. Differential privacy. In *Proceedings of the 33rd International Conference on Automata, Languages and Programming - Volume Part II*, ICALP'06, pages 1–12, 2006.

[4] Mikhail J Atallah, Florian Kerschbaum, and Wenliang Du. Secure and private sequence comparisons. In *Proceedings of the 2003 ACM workshop on Privacy in the electronic society*, pages 39–44. ACM, 2003.

[5] Marina Blanton and Mehrdad Aliasgari. Secure outsourcing of DNA searching via finite automata. In *IFIP Annual Conference on Data and Applications Security and Privacy*, pages 49–64. Springer, 2010.

[6] Juan Ramón Troncoso-Pastoriza, Stefan Katzenbeisser, and Mehmet Celik. Privacy preserving error resilient DNA searching through oblivious automata. In *Proceedings of the 14th ACM conference on Computer and communications security*, pages 519–528. ACM, 2007.

[7] Hiroki Sudo, Masanobu Jimbo, Koji Nuida, and Kana Shimizu. Secure Wavelet Matrix: Alphabet-Friendly Privacy-Preserving String Search. *BioRxiv*, page 085647, 2016.

[8] Md Safiur Rahman Mahdi, Mohammad Zahidul Hasan, and Noman Mohammed. Secure sequence similarity search on encrypted genomic data. In *2017 IEEE/ACM International Conference on Connected Health: Applications, Systems and Engineering Technologies (CHASE)*, pages 205–213. IEEE, 2017.

[9] Md Safiur Rahman Mahdi, Md Momin Al Aziz, Dima Alhadidi, and Noman Mohammed. Secure similar patients query on encrypted genomic data. *IEEE journal of biomedical and health informatics*, 23(6):2611–2618, 2018.

[10] Yu Ishimaki, Hiroki Imabayashi, Kana Shimizu, and Hayato Yamana. Privacy-preserving string search for genome sequences with FHE bootstrapping optimization. In *2016 IEEE International Conference on Big Data (Big Data)*, pages 3989–3991. IEEE, 2016.

[11] Kana Shimizu, Koji Nuida, and Gunnar Rätsch. Efficient privacy-preserving string search and an application in genomics. *Bioinformatics*, 32(11):1652–1661, 2016.

[12] Richard Durbin. Efficient haplotype matching and storage using the positional burrows–wheeler transform (pbwt). *Bioinformatics*, 30(9):1266–1272, 2014.

[13] Y. Yamada, K. Rohloff, and M. Oguchi. Homomorphic encryption for privacy-preserving genome sequences search. In *2019 IEEE International Conference on Smart Computing (SMARTCOMP)*, pages 7–12, 2019.

[14] Tsung-Ting Kuo, Xiaoqian Jiang, Haixu Tang, XiaoFeng Wang, Tyler Bath, Diyue Bu, Lei Wang, Arif Harmanci, Shaojie Zhang, Degui Zhi, et al. idash secure genome analysis competition 2018: blockchain genomic data access logging, homomorphic encryption on gwas, and dna segment searching. *BMC Medical Genomics*, 13(Suppl 7), 2020.

[15] Katerina Sotiraki, Esha Ghosh, and Hao Chen. Privately computing set-maximal matches in genomic data. *BMC Medical Genomics*, 13(7):1–8, 2020.

[16] Oded Goldreich, Silvio Micali, and Avi Wigderson. How to play any mental game, or a completeness theorem for protocols with honest majority. In *Providing Sound Foundations for Cryptography: On the Work of Shafi Goldwasser and Silvio Micali*, pages 307–328. 2019.

[17] Martin Farach, Paolo Ferragina, and Shanmugavelayutham Muthukrishnan. Overcoming the memory bottleneck in suffix tree construction. In *Proceedings 39th FOCS*, pages 174–183. IEEE, 1998.

[18] Sandeep Tata, Richard A Hankins, and Jignesh M Patel. Practical suffix tree construction. In *Proceedings of the 13th intl. conf. VLDB*, pages 36–47, 2004.

[19] Yuanyuan Tian, Sandeep Tata, Richard A Hankins, and Jignesh M Patel. Practical methods for constructing suffix trees. *The VLDB Journal*, 14(3):281–299, 2005.

[20] Benjarath Phoophakdee and Mohammed J Zaki. Genome-scale disk-based suffix tree indexing. In *SIGMOD int. conf. on Management of data*, pages 833–844. ACM, 2007.

[21] Amol Ghoting and Konstantin Makarychev. Serial and parallel methods for i/o efficient suffix tree construction. In *Proceedings of the 2009 ACM SIGMOD Int'l Conference on Management of data*, pages 827–840. ACM, 2009.

[22] Essam Mansour, Amin Allam, Spiros Skiadopoulos, and Panos Kalnis. ERA: efficient serial and parallel suffix tree construction for very long strings. *Proceedings of the VLDB*, 5(1):49–60, 2011.

[23] Matteo Comin and Montse Farreras. Parallel continuous flow: a parallel suffix tree construction tool for whole genomes. *Jrnl. of Comp. Biology*, 21(4):330–344, 2014.

[24] Guanghui et al. Zhu. DGST: Efficient and scalable suffix tree construction on distributed data-parallel platforms. *Parallel Computing*, 87:87–102, 2019.

[25] Julian Shun and Guy E Blelloch. A simple parallel cartesian tree algorithm and its application to parallel suffix tree construction. *ACM TOPC*, 1(1):8, 2014.

[26] Patrick Flick and Srinivas Aluru. Parallel construction of suffix trees and the all-nearest-smaller-values problem. In *2017 IEEE International Parallel and Distributed Processing Symposium (IPDPS)*, pages 12–21. IEEE, 2017.

[27] Freeson Kaniwa, Otlhapile Dinakenyane, and Venu Madhav Kuthadi. Parallel algorithm for indexing large dna sequences using mapreduce on hadoop. In *2017 IEEE International Conference on Bioinformatics and Biomedicine (BIBM)*, pages 1576–1582. IEEE, 2017.

[28] Yunhao Li, Jiahui Jin, Runqun Xiong, and Junzhou Luo. A distributed approach for constructing generalized suffix tree on spark by using optimized elastic range algorithm. In *2017 Fifth International Conference on Advanced Cloud and Big Data (CBD)*, pages 117–122. IEEE, 2017.

[29] M. J. Mišić, D. V. Nikolov, J. Ž. Protić, and M. V. Tomašević. Parallelization of gst algorithm for source code similarity detection. In *2016 24th Telecommunications Forum (TELFOR)*, pages 1–4, 2016.

[30] Alan Hodgkinson and Adam Eyre-Walker. Human triallelic sites: evidence for a new mutational mechanism? *Genetics*, 184(1):233–241, 2010.

[31] Donald E Knuth. Sorting and searching. 1973.

[32] Dan Gusfield. Algorithms on stings, trees, and sequences: Computer science and computational biology. *Acm Sigact News*, 28(4):41–60, 1997.

[33] E. Ukkonen. Online construction of suffixtrees. *Algorithmica*, 14(3):249–260, 1995.

[34] Andrew Chi-Chih Yao. Protocols for secure computations. In *FOCS*, volume 82, pages 160–164, 1982.
